# Small Bowel Volvulus Diagnosed and Treated Using Intraoperative Angiography in a Hybrid Operating Room: A Case Report

**DOI:** 10.70352/scrj.cr.26-0410

**Published:** 2026-10-16

**Authors:** Hirotaka Gambo, Harufumi Maki, Motomu Tanaka, Masashi Momiyama, Kentaro Nakajima, Atsuki Nagao, Marie Osawa, Shouichi Satou

**Affiliations:** 1 Department of Surgery, NTT Medical Center Tokyo, Tokyo, Japan; 2 Department of Radiology, NTT Medical Center Tokyo, Tokyo, Japan

**Keywords:** small bowel volvulus, hybrid operating room, abdominal angina, angiography, alprostadil

## Abstract

**INTRODUCTION:**

Small bowel volvulus is a surgical emergency that may cause mesenteric ischemia; however, diagnosis may be difficult when bowel perfusion appears preserved.

**CASE PRESENTATION:**

An 84-year-old man presented with recurrent postprandial abdominal pain and melena 6 months after a total pharyngolaryngectomy with a jejunal free flap reconstruction. Extensive endoscopic evaluations, including capsule endoscopy, revealed no bleeding source. Contrast-enhanced CT revealed the whirl sign at the root of the small-bowel mesentery with the narrowing of the superior mesenteric artery (SMA), while bowel wall enhancement was preserved. Because the symptoms recurred after resuming oral intake, a hybrid surgery combined with angiography was planned for both diagnostic and therapeutic purposes. The SMA angiography demonstrated significant stenosis without apparent bowel hypoperfusion. After the intra-arterial administration of alprostadil, distal mesenteric perfusion deteriorated on the angiography, and laparoscopic exploration revealed ischemic changes consistent with small bowel volvulus. Because laparoscopic reduction was difficult, an open reduction was performed. Repeat angiography confirmed the resolution of the SMA narrowing and the restoration of distal flow. The patient’s symptoms resolved completely.

**CONCLUSIONS:**

The hybrid surgery enabled a functional assessment of mesenteric hemodynamics and immediate surgical treatment during a single procedure. It may be useful in selected patients with small bowel volvulus who have preserved bowel perfusion at rest but recurrent symptoms suggestive of intermittent mesenteric ischemia, and in whom the indication for surgical detorsion remains uncertain.

## Abbreviations

CA
: celiac artery
IMA
: inferior mesenteric artery
SMA
: superior mesenteric artery
SMV
: superior mesenteric vein

## INTRODUCTION

Small bowel volvulus is an acute abdominal condition caused by the rotation of the small intestine around the mesenteric axis and often requires urgent surgical intervention.^1,2)^ The causes of small bowel volvulus are broadly classified into congenital factors, such as intestinal malrotation or fixation abnormalities; secondary factors including postoperative adhesions, tumors, or Meckel’s diverticulum; and primary volvulus. Although emergent surgery is recommended for patients with bowel ischemia or obstruction, the optimal management of patients with suspected small bowel volvulus and preserved bowel perfusion remains controversial.^3)^ In these patients, determining whether the symptoms are caused by small bowel volvulus or another condition may be difficult. Therefore, an additional functional assessment of mesenteric blood flow may be useful.

Hybrid surgery, which integrates intraoperative angiography and surgical procedures, has been reported to be effective in trauma and vascular surgery.^4,5)^ However, reports of its application in gastrointestinal surgery—particularly for simultaneous intraoperative angiographic diagnosis and treatment—are extremely limited.^6)^

We report a case in which intraoperative angiography performed in a hybrid operating room allowed real-time evaluation of mesenteric hemodynamics, followed by immediate surgical treatment of small bowel volvulus.

## CASE PRESENTATION

An 84-year-old man presented with abdominal pain. Eight months earlier, he had undergone a total pharyngolaryngectomy with a free jejunal reconstruction, a right thyroid lobectomy, and the creation of a permanent tracheostoma for hypopharyngeal cancer. He had experienced abdominal pain accompanied by hematochezia for 2 months before admission. Contrast-enhanced CT at the initial admission demonstrated a whirl sign with the rotation of the small bowel around the SMA and the SMV, accompanied by mesenteric edema and suspected SMV thrombosis, without evidence of bowel ischemia. Upper and lower gastrointestinal endoscopy revealed no obvious abnormalities, and capsule endoscopy of the small intestine also showed no significant findings. Although his symptoms temporarily improved and he was discharged, he was readmitted 1 month later because of a recurrence of the same symptoms.

His medical history included a silent old cerebral infarction, asthma, hypertension, dyslipidemia, hyperuricemia, and Barrett’s esophageal adenocarcinoma treated endoscopically. On readmission, his height was 175.0 cm and body weight was 51.2 kg (BMI 16.7 kg/m^2^). The abdomen was flat and soft with mild diffuse tenderness but no rebound tenderness. A digital rectal examination revealed no palpable mass, and hard brown stool was noted. Laboratory findings were as follows: white blood cell count 2.3 × 10^3^/μL, hemoglobin 8.3 g/dL, aspartate aminotransferase 16 U/L, alanine aminotransferase 10 U/L, lactate dehydrogenase 190 U/L, creatine kinase 67 U/L, C-reactive protein ≤0.30 mg/dL, activated partial thromboplastin time 31.8 s, and prothrombin time–international normalized ratio 1.00. Abdominal ultrasonography demonstrated no obvious stenosis of the SMA. On readmission, contrast-enhanced CT showed a small bowel volvulus, with a whirl sign at the root of the small-bowel mesentery and the narrowing of the SMA (**Fig. 1A**). The mesentery appeared edematous, but the bowel-wall enhancement was preserved, and there was no obvious bowel obstruction. A follow-up CT obtained 5 days after admission still suggested a small bowel volvulus, but the previously observed severe SMA narrowing had resolved, and the bowel wall enhancement remained preserved without evidence of venous congestion (**Fig. 1B**).

**Fig. 1:**
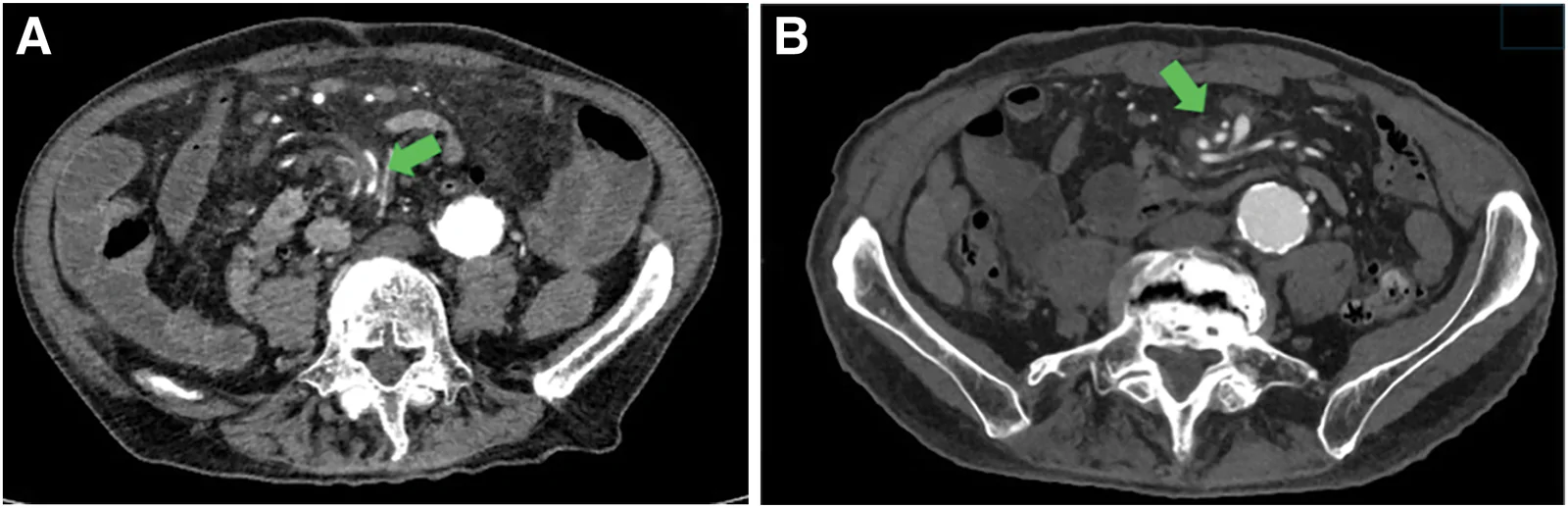
(**A**) Contrast-enhanced CT on readmission showed that the small bowel mesentery appeared edematous. A whirl sign was observed at the root of the small bowel mesentery and narrowing of the SMA (arrow). (**B**) Follow up contrast-enhanced CT suggested persistent small bowel volvulus, with resolved the SMA narrowing and preserved bowel enhancement (arrow). SMA, superior mesenteric artery

After the improvement of abdominal pain and hematochezia, oral intake was resumed. However, the abdominal pain recurred after meals. Although there was no obvious bowel obstruction, small bowel volvulus was considered a relative indication for surgery as the likely cause of the recurrent abdominal pain and hematochezia. Because mesenteric ischemia resembling abdominal angina was also suspected, an intraoperative assessment of mesenteric blood flow was planned in a hybrid operating room.

The procedure was performed in a hybrid operating room. A vascular sheath was inserted via the right femoral artery. Angiography of the CA demonstrated no apparent anatomical variations, and antegrade blood flow in the gastroduodenal artery was preserved. Subsequent angiography of the SMA revealed significant stenosis (**Fig. 2**). After the intra-arterial administration of alprostadil, SMA angiography demonstrated markedly reduced blood flow distal to the stenotic segment, with clearly decreased visualization of the vasa recta (**Fig. 3**). In addition, a to-and-fro flow from the IMA was observed, suggesting the presence of collateral circulation originating from the IMA. Laparoscopic exploration was then initiated. Intra-abdominal observation revealed torsion centered at the root of the small-bowel mesentery. A total of 0.25 mg of indocyanine green was administered intra-arterially through the SMA. Despite the direct intra-arterial administration, visualization of intestinal perfusion required approximately 2 minutes and 30 seconds before reduction of the volvulus (**Fig. 4**). Although laparoscopic detorsion was attempted, reduction of the volvulus was difficult; therefore, the procedure was converted to an open laparotomy. The small intestine was found to be twisted 270°counterclockwise around the root of the mesentery. The volvulus was manually reduced by clockwise rotation. Pulsation of the SMA was confirmed by palpation. As before the detorsion, 0.25 mg of indocyanine green was administered intra-arterially through the SMA after detorsion. In contrast to the delayed fluorescence observed before detorsion, intestinal perfusion was visualized within approximately 30 seconds, and the abdomen was subsequently closed. Repeat SMA angiography demonstrated the resolution of the stenosis. Additional intra-arterial administration of alprostadil did not reproduce the stenosis. Narrowings were noted near the origins of 2 intestinal branches of the SMA; however, these were judged suitable for observation (**Fig. 5**). The interval from the insertion of the femoral arterial sheath to the completion of the final angiography was 198 minutes, and the total operative time was 222 minutes.

**Fig. 2:**
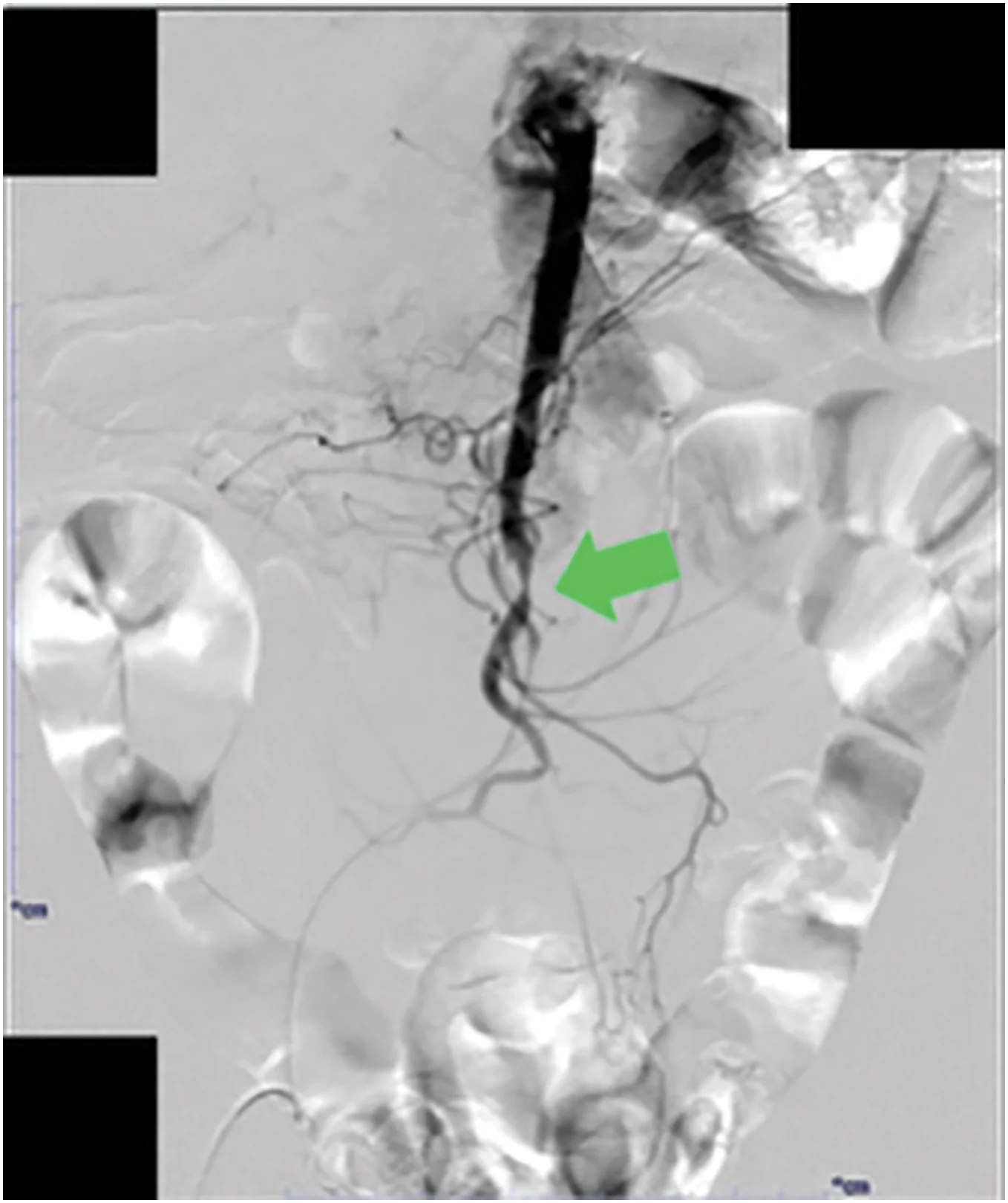
Intraoperative angiography demonstrated significant stenosis of the SMA (arrow). SMA, superior mesenteric artery

**Fig. 3:**
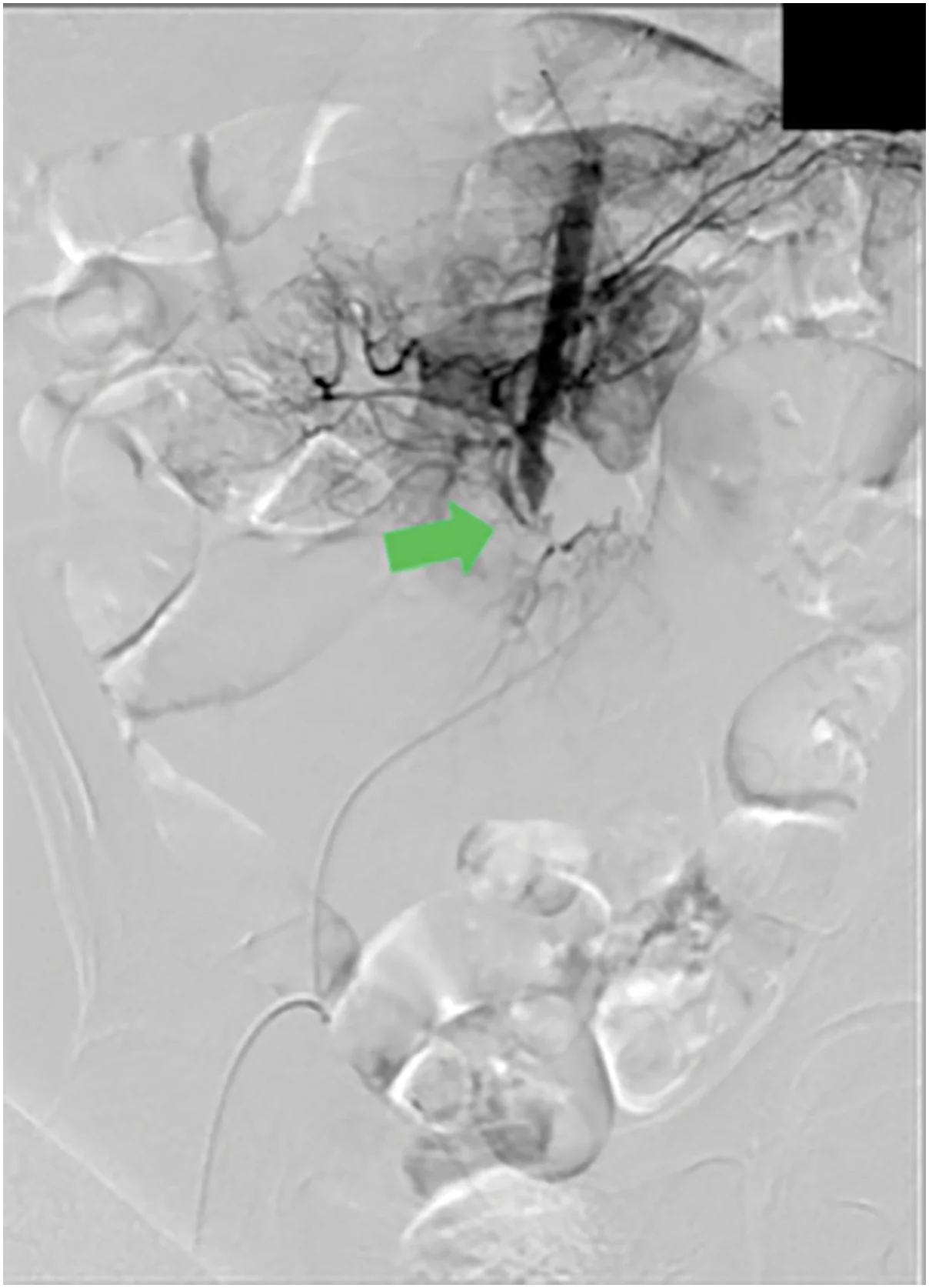
The stenosis of the SMA became more pronounced and progressed to complete occlusion after administration of alprostadil (arrow). SMA, superior mesenteric artery

**Fig. 4:**
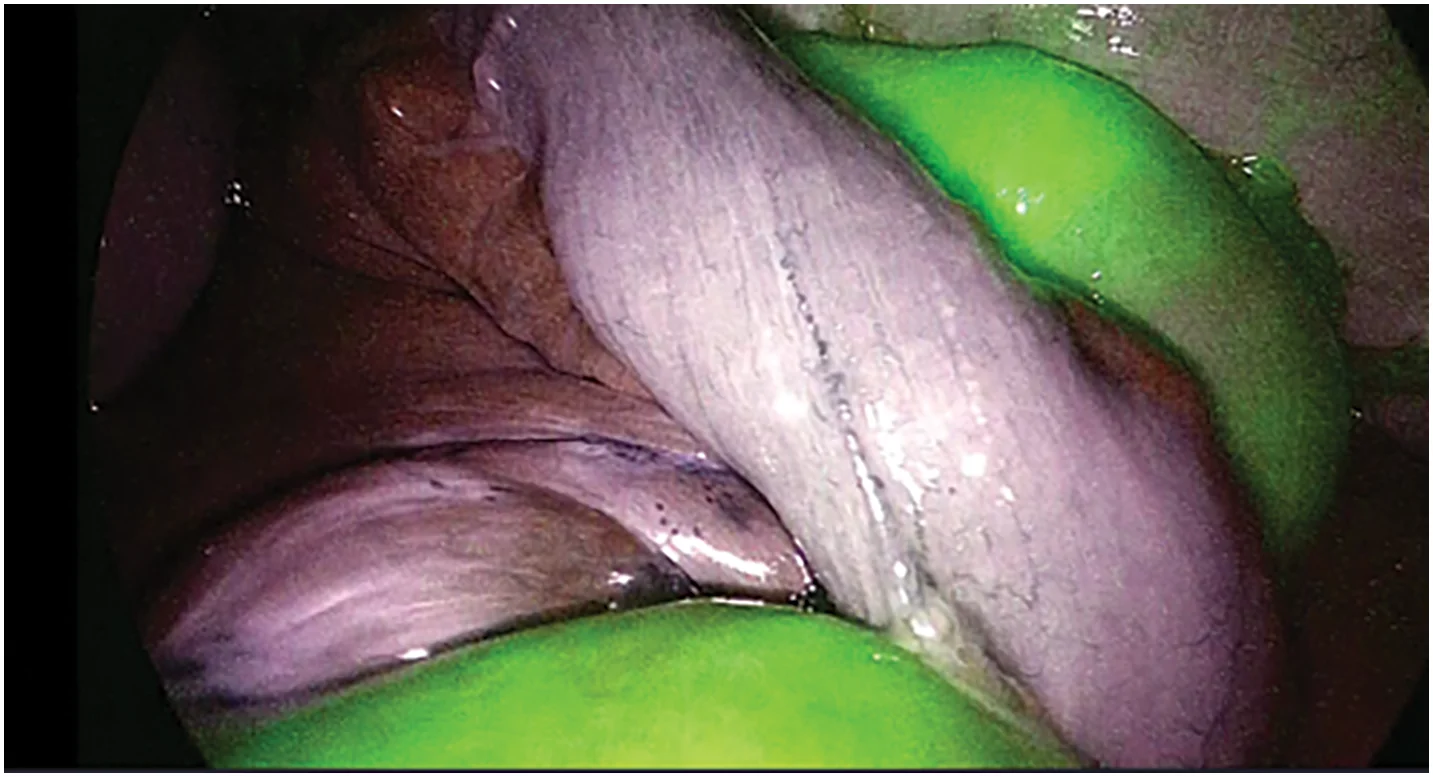
Intraoperative ICG fluorescence imaging before detorsion of the small bowel volvulus. Following intra-arterial administration of ICG through the SMA, bowel segments with preserved perfusion showed green fluorescence. In contrast, bowel segments with impaired perfusion showed little or no fluorescence, demonstrating heterogeneous intestinal perfusion. ICG, indocyanine green; SMA, superior mesenteric artery

**Fig. 5:**
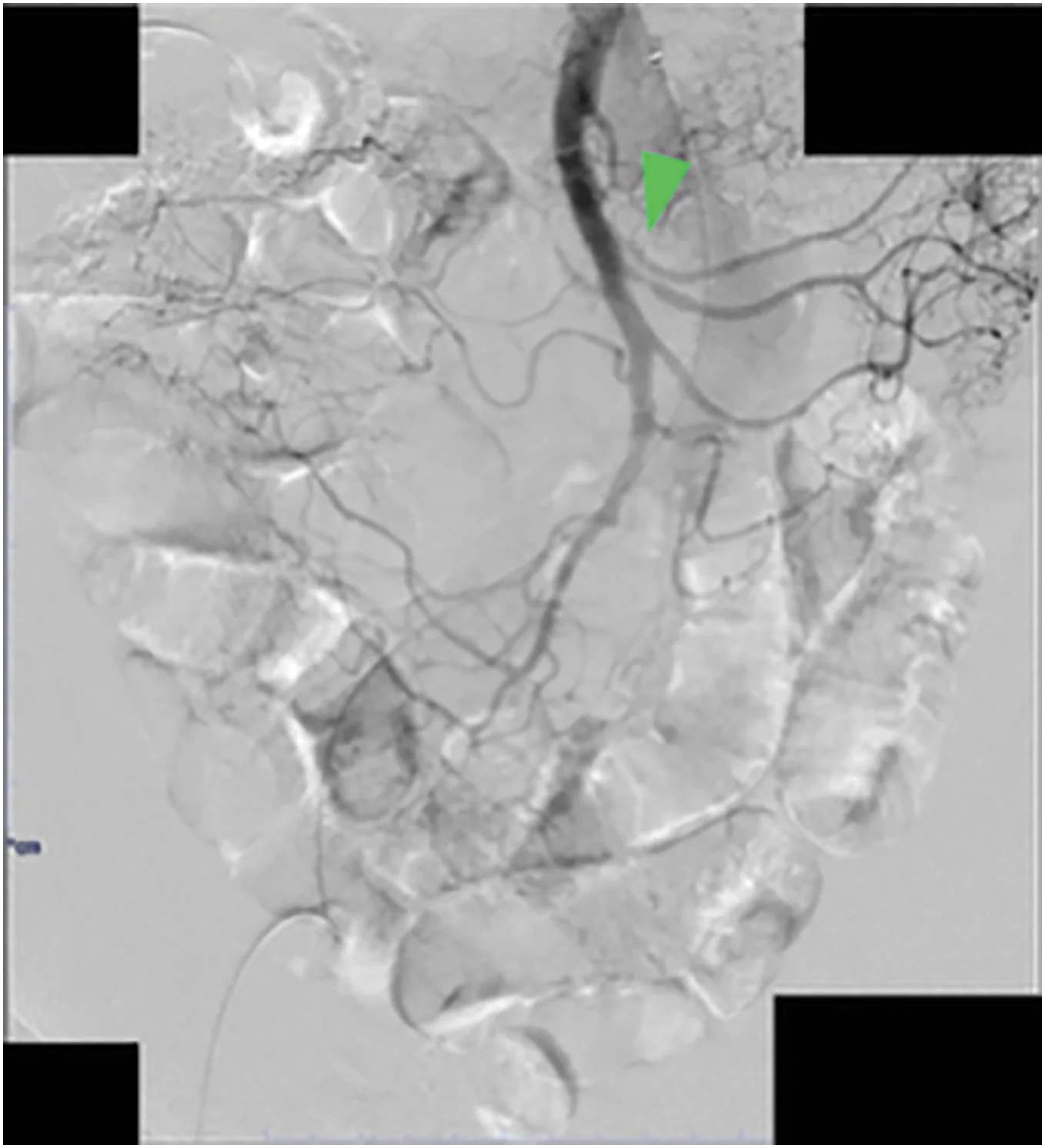
The angiography after reduction of the volvulus demonstrated resolution of the bowel perfusion. Narrowings were noted near the origins of the 2 branches of the SMA, which require no intervention (arrow head). SMA, superior mesenteric artery

The postoperative course was uneventful. The patient had no recurrence of hematochezia or abdominal pain and was discharged on POD 12. He has been followed up regularly in the outpatient clinic and remains asymptomatic. At 3 months after surgery, no recurrence of the symptoms has been observed.

## DISCUSSION

To our knowledge, there have been no previous reports describing a hybrid surgery for small bowel volvulus. In the present case, intraoperative angiography provided functional information regarding mesenteric perfusion that could not be obtained from static imaging alone.

Small bowel volvulus is a relatively rare condition, and the whirl sign is considered a characteristic imaging feature.^7,8)^ However, similar findings may be observed in postoperative anatomical changes or internal hernias, and the whirl sign alone does not always indicate an acute volvulus requiring emergency surgery.^9)^ In the present case, a typical whirl sign was identified on CT imaging; however, intestinal perfusion and SMA blood flow were preserved, and the patient’s symptoms had spontaneously resolved. Therefore, the clinical findings were insufficient to justify an emergency surgical intervention, and conservative management was initially selected. These findings suggested that anatomical imaging alone was insufficient to determine whether clinically significant mesenteric ischemia was present, and an additional functional assessment of mesenteric perfusion was considered necessary.

Abdominal angina generally results from chronic intestinal ischemia caused by the stenosis or occlusion of major visceral arteries, including the CA, the SMA, and the IMA, and typically presents with postprandial abdominal pain.^10)^ Diagnostic evaluation of mesenteric blood flow may include abdominal ultrasonography, CT, and MRA, which can demonstrate the stenosis or occlusion of major visceral arteries.^11)^ Catheter-based angiography is considered useful for establishing the definitive diagnosis.^11)^ In general, angiography is first performed for diagnostic purposes, followed by surgical revascularization when indicated. In our case, after resumption of oral intake, abdominal pain and hematochezia recurred, whereas the symptoms improved again after fasting. Based on this clinical course, a mechanism similar to abdominal angina was suspected, in which increased intestinal metabolic demand after food intake results in increased mesenteric blood flow requirements.^12,13)^ Therefore, intraoperative angiography in a hybrid operating room would be useful to allow real-time diagnosis. An alternative strategy would have been preoperative catheter angiography followed by surgery if indicated. However, angiography alone cannot treat mechanical volvulus, whereas surgical exploration alone cannot objectively evaluate mesenteric hemodynamics. The principal value of the hybrid operating room in the present case was the ability to integrate functional hemodynamic assessment and definitive surgical treatment during a single procedure. The hybrid operating room enabled sequential angiographic assessment, laparoscopic confirmation, and immediate open detorsion, and subsequent angiographic confirmation of improved SMA blood flow.

Alprostadil has been used to enhance portal-phase contrast during transarterial portography via the SMA.^14)^ It has pharmacological effects that reduce peripheral vascular resistance.^15)^ In addition, intra-arterial administration of alprostadil has been reported to dilate the SMA in patients with non-occlusive mesenteric ischemia.^16)^ In the present case, alprostadil was administered intra-arterially to pharmacologically augment mesenteric perfusion and evaluate the hemodynamic significance of the SMA narrowing. However, distal SMA blood flow unexpectedly disappeared with clearly decreased visualization of the vasa recta after intra-arterial administration of alprostadil. Even if adequate distal perfusion had been demonstrated after alprostadil administration, surgical exploration would still have been indicated because the recurrent postprandial symptoms and persistent radiological findings suggested intermittent mechanical volvulus.

One possible explanation for this atypical response is the following hemodynamic change. Although the structural stenosis itself would not be improved by alprostadil, the drug may have reduced peripheral vascular resistance in the proximal SMA branches, particularly in the region of the inferior pancreaticoduodenal artery. Consequently, contrast inflow into these proximal branches may have increased relatively, resulting in reduced perfusion distal to the stenotic segment and eventual loss of visualization of the distal SMA. However, the precise mechanism underlying this phenomenon remains unclear and warrants further investigation.

## CONCLUSIONS

In the present case, intraoperative angiography and intra-arterial infusion of alprostadil enabled functional assessment of mesenteric hemodynamics and provided additional information regarding mesenteric perfusion, while surgical detorsion simultaneously relieved the SMA stenosis caused by the volvulus.

These findings suggest that hybrid surgery combining intraoperative angiography with surgical treatment may represent a useful option in selected patients with small bowel volvulus and suspected intermittent mesenteric ischemia, particularly in those with recurrent postprandial symptoms despite preserved bowel enhancement on CT, in whom the indication for emergent surgery remains uncertain.

## DECLARATIONS

### Availability of data and materials

Data are available from the corresponding author upon reasonable request.

## References

[1] Shyam DC, Shyam RC. Small bowel volvulus in adult: a review of literature. Eur J Med Health Sci 2020; 2: 288.

[2] Fan S, Wang J, Li Y, et al. Clinical analysis of acute small bowel volvulus in the late postoperative period following gastrectomy. Int J Clin Exp Med 2018; 11: 11096–103.

[3] Li X, Zhang J, Li B, et al. Diagnosis, treatment and prognosis of small bowel volvulus in adults: a monocentric summary of a rare small intestinal obstruction. PLoS One 2017; 12: e0175866. 10.1371/journal.pone.0175866 PMC5398554 28426721

[4] Kataoka Y, Minehara H, Kashimi F, et al. Hybrid treatment combining emergency surgery and intraoperative interventional radiology for severe trauma. Injury 2016; 47: 59–63. 10.1016/j.injury.2015.09.022 26508437

[5] Bunya N, Harada K, Kuroda Y, et al. The effectiveness of hybrid treatment for sever multiple trauma: a case of multiple trauma for damage control laparotomy and thoracic endovascular repair. Int J Emerg Med 2017; 10: 18. 10.1186/s12245-017-0145-8 PMC5459779 28585119

[6] Kawachi J, Ogino H, Shimoyama R, et al. Intraoperative angiography with indocyanine green injection for precise localization and resection of small bowel bleeding. Acute Med Surg 2020; 7: e549. 10.1002/ams2.549 PMC7426192 32817800

[7] Iwuagwu O, Deans GT. Small bowel volvulus: a review. J R Coll Surg Edinb 1999; 44: 150–5. 10372482

[8] Rowsom JT, Sullivan SN, Girvan DP. Midgut volvulus in the adult. J Clin Gastroenterol 1987; 9: 212–6. 10.1097/00004836-198704000-00021 3571896

[9] Gollub MJ, Yoon S, Smith LM, et al. Does the CT whirl sign really predict small bowel volvulus? Experience in an oncologic population. J Comput Assist Tomogr 2006; 30: 25–32. 10.1097/01.rct.0000191680.28344.f0 16365568

[10] Terlouw LG, Moelker A, Abrahamsen J, et al. European guidelines on chronic mesenteric ischaemia - joint United European Gastroenterology, European Association for Gastroenterology, Endoscopy and Nutrition, European Society of Gastrointestinal and Abdominal Radiology, Netherlands Association of Hepatogastroenterologists, Hellenic Society of Gastroenterology, Cardiovascular and Interventional Radiological Society of Europe, and Dutch Mesenteric Ischemia Study group clinical guidelines on the diagnosis and treatment of patients with chronic mesenteric ischaemia. United European Gastroenterol J 2020; 8: 371–95. 10.1177/2050640620916681 PMC7226699 32297566

[11] Pillai AK, Kalva SP, Hsu SL, et al. Quality improvement guidelines for mesenteric angioplasty and stent placement for the treatment of chronic mesenteric ischemia. J Vasc Interv Radiol 2018; 29: 642–7. 10.1016/j.jvir.2017.11.024 29574024

[12] Abdullah E, Lee JB, Notay K, et al. Postprandial superior mesenteric artery blood flow is related to changes in peripheral pulse wave harmonics and heart rate: implications for wearable technology. J Appl Physiol (1985) 2021; 131: 681–8. 10.1152/japplphysiol.00903.2020 34264128

[13] Sidery MB, Macdonald IA, Blackshaw PE. Superior mesenteric artery blood flow and gastric emptying in humans and the differential effects of high-fat and high-carbohydrate meals. Gut 1994; 35: 186–90. 10.1136/gut.35.2.186 PMC1374492 8307468

[14] Ito T, Itoshima T, Ukida M, et al. Enhanced visualization of the portal vein system in superior mesenteric arterial portography using prostaglandin E1. Acta Med Okayama 1982; 36: 291–7. 10.18926/AMO/30653 6890302

[15] Ono Pharmaceutical Co. Ltd. Prostaglandin E1 (alprostadil) injection [package insert]. Osaka: Ono Pharmaceutical Co., Ltd.; 2024.

[16] Ogi K, Sanui M, Iizuka Y, et al. Successful treatment of nonocclusive mesenteric ischemia after aortic valve replacement with continuous arterial alprostadil infusion: a case report. Int J Surg Case Rep 2017; 35: 8–11. 10.1016/j.ijscr.2017.03.037 PMC5394210 28414997

